# Should we switch to tenecteplase for all ischemic strokes? Evidence and logistics

**DOI:** 10.1177/17474930241307098

**Published:** 2025-01-06

**Authors:** Keith W Muir

**Affiliations:** School of Cardiovascular & Metabolic Health, University of Glasgow, Queen Elizabeth University Hospital, Glasgow, UK

**Keywords:** Acute stroke therapy, rtPA, thrombolysis, tPA, tenecteplase, acute

## Abstract

Recent clinical trials provide robust evidence of non-inferiority of tenecteplase 0.25 mg/kg over alteplase 0.9 mg/kg in acute ischemic stroke treated within 4.5 h of time last known well. Aggregate data meta-analysis suggests likely superiority of tenecteplase with respect to excellent (modified Rankin Scale 0 or 1) outcomes at 90 days. Less complex single intravenous bolus administration of tenecteplase brings significant logistical benefits compared to alteplase. Real-world implementation data demonstrate reduced door-to-needle and door-to-puncture times, and potentially improved clinical outcomes. Avoiding the need for infusion pumps and monitoring reduces resource requirements and facilitates inter-hospital transfer. Guidelines favor tenecteplase over alteplase due to its logistical advantages. Transitioning services to tenecteplase requires consideration of education and training for all relevant staff (medical, nursing, pharmacy) and should address physician concerns. Use of stroke-specific tenecteplase 25 mg dose vials is strongly preferable to minimize the chance of dosing errors that might arise from use of cardiac-dose tenecteplase. Some off-label uses of alteplase are supported by positive randomized controlled trial data (wake-up and unknown onset stroke, and imaging-supported late window use 4.5–9 h after onset) while equivalent data for tenecteplase are less conclusive. Trial data comparing tenecteplase to control give relevant safety data for both wake-up / unknown onset stroke and for late time windows, and some efficacy data favor tenecteplase in a late time window. Given the weight of evidence for biologically similar efficacy and safety of tenecteplase 0.25 mg/kg, and potential for dosing errors, retention of alteplase for off-label indications should not be recommended.

Thrombolytic drug treatment remains the most widely accessible option for reperfusion in acute ischemic stroke, either alone or as bridging therapy for those eligible for, and able to access, endovascular thrombectomy. New evidence indicates that a potentially important improvement in patient care can be achieved with transitioning to tenecteplase as the thrombolytic agent of choice.

## Trial evidence supporting tenecteplase over alteplase

Following on from a series of small phase II trials that compared tenecteplase to alteplase, a series of recent randomized controlled trials (RCTs) have provided clear evidence for the non-inferiority,^1–4^ and likely superiority,^5^ of tenecteplase 0.25 mg/kg over alteplase 0.9 mg/kg for acute ischemic stroke treated within 4.5 h of being last known well. RCTs that compared tenecteplase with alteplase are detailed in Table 1. Following small trials that focused on biomarker outcomes or safety,^6–8^ the Norwegian NOR-TEST trial compared a dose of tenecteplase of 0.4 mg/kg against alteplase,^9^ but was inconclusive. NOR-TEST was compromised by the inclusion of a high proportion of stroke mimics and minor strokes, but the subsequent NOR-TEST 2A trial was stopped on safety grounds and concluded that this dose was associated with excess risk of intracerebral hemorrhage.^12^ The EXTEND-IA TNK 2 trial^15^ compared 0.4 mg/kg tenecteplase against 0.25 mg/kg tenecteplase and found no difference in outcomes among a selected population undergoing thrombectomy, although numerically higher symptomatic intracerebral hemorrhage (SICH) risk. Further investigation of the 0.4 mg/kg dose has not been undertaken following NOR-TEST 2A.

**Table 1.** Randomized controlled clinical trials comparing tenecteplase with alteplase in acute stroke within 4.5 h from time last known well.

	Year	Tenecteplase dose	n	Primary outcome
Haley et al.^6^	2010	0.1, 0.25, 0.4 mg/kg	112	Ph2 safety / efficacy
Parsons et al.^7^	2012	0.1, 0.25 mg/kg	75	Ph2 reperfusion
ATTEST^8^	2015	0.25 mg/kg	104	Ph2 penumbra salvage
NOR-TEST^9^	2017	0.4 mg/kg	1100	mRS
EXTEND-IA TNK^10^	2018	0.25 mg	202	Reperfusion
TRACE^* 11^	2021	0.1, 0.25, 0.32 mg/kg	236	Ph2 safety / efficacy
NOR-TEST-2A^12^	2022	0.4 mg/kg	216	Safety (non-inferiority)
TASTE-A^13^	2022	0.25 mg/kg	104	Reperfusion
ACT^1^	2022	0.25 mg/kg	1600	Non-inferiority
TRACE-2^* 3^	2023	0.25 mg/kg	1430	Non-inferiority
TASTE^4^	2024	0.25 mg/kg	680	Non-inferiority
ATTEST-2^2^	2024	0.25 mg/kg	1777	Non-inferiority
ORIGINAL^14^	2024	0.25 mg/kg	1468	Non-inferiority

* TRACE and TRACE-2 used a biocopy tenecteplase molecule currently available only in China.

The EXTEND-IA TNK trial was undertaken in patients undergoing thrombectomy and found a significantly higher proportion of tenecteplase 0.25 mg/kg-treated patients to have reperfused at the time of initial angiographic assessment.^10^

**Figure 1.**
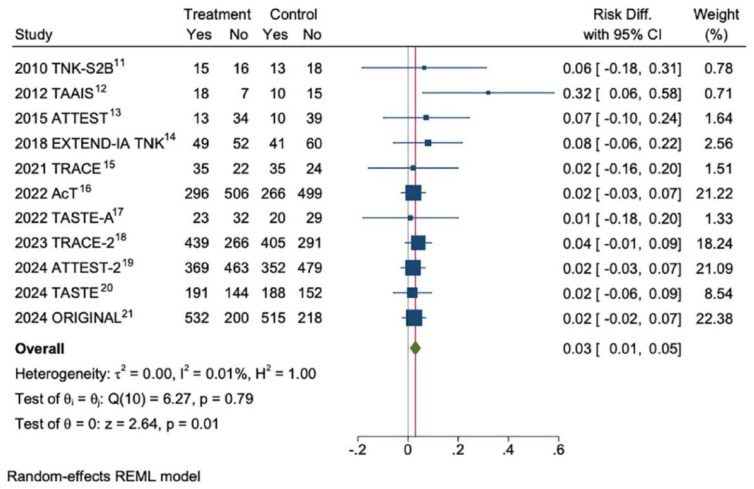
Random-effects meta-analysis of aggregate data from RCTs that compared tenecteplase 0.25 mg/kg against alteplase < 4.5 h from time last known well. Source: Campbell.^16^

The trials that compared tenecteplase 0.25 mg/kg directly against alteplase as standard of care involved in total over 7500 patients. Predefined non-inferiority margins were met in AcT,^1^ ORIGINAL, TRACE-2^3^ and ATTEST-2.^2^ The TASTE trial stopped early due to the presentation of evidence from these other RCTs and was therefore underpowered for its non-inferiority analysis.^4^ While no individual trial was able to demonstrate superiority, aggregate data meta-analysis indicates increased likelihood of excellent functional outcome (mRS 0–1 at 3 months, risk ratio in favor of tenecteplase 1.05, 95% confidence interval 1.01–1.10), an absolute benefit of around 3%–5% depending on the meta-analysis techniques employed (Figure 1).^5^

Even prior to the most recent trial data, European Stroke Organization guidelines recommended tenecteplase 0.25 mg/kg as an alternative to alteplase in the < 4.5 h time window, and favored tenecteplase over alteplase for general use due to easier administration, and for those with large vessel occlusion, based on the EXTEND-IA TNK findings.^17^ Subsequent trial data have not found evidence of superiority in this subgroup, however.^2,18^ The emerging consensus based on new trial data is in favor of tenecteplase as the standard of care thrombolytic agent in acute stroke.^19^

## Logistic benefits of tenecteplase

A major driver for change is the potential impact of tenecteplase on logistics and workflow around thrombolysis. Both tenecteplase and alteplase are tissue-type plasminogen activators. The tenecteplase molecule has three amino acid modifications that have the consequences of enhanced fibrin specificity and greater resistance to inactivation by plasminogen activator inhibitor type 1, yielding a longer half-life for tenecteplase.^20^ Administration of tenecteplase is consequently possible as a short intravenous bolus over a few seconds. In contrast, the short circulating half-life of alteplase requires the more demanding regime of a 10% bolus followed by 1-h infusion of the remaining 90% of the dose. The logistics of thrombolytic treatment in the context of both the in-hospital and between-hospital movement of patients that are commonly required are therefore much easier with tenecteplase. Bolus administration additionally avoids the potential compromise of thrombolytic effect due to delays between bolus and infusion start with alteplase. The circulating levels of alteplase reduce by half in only 5 min,^21^ an interval that may be exceeded in a majority of patients treated with alteplase.^22^ In countries and regions that elected to move to routine tenecteplase use prior to the recent RCTs, benefits observed in real-world implementation of tenecteplase include shortened door-to-needle times, easier thrombolytic delivery in locations, such as scanner rooms, and reduced onset-to-groin puncture times among those eligible for endovascular thrombectomy.^23,24^

The case for switching standard thrombolytic from alteplase to tenecteplase is therefore clear. What are the potential barriers to implementing tenecteplase as the standard of care thrombolytic agent for stroke?

## Potential for tenecteplase dosing errors

One potential concern is that of dosing and administration errors. The stroke treatment dose of tenecteplase is half that of the dose for treatment of acute myocardial infarction (MI; 0.5 mg/kg). Since tenecteplase at the higher dose of 0.4 mg/kg was significantly more hazardous than alteplase,^12^ there are potential risks of giving a dose intended for acute MI. While the manufacture and wide availability of separately packaged tenecteplase 25 mg vials specifically intended for stroke treatment mitigates the risk, nonetheless both MI and stroke treatment supplies are likely to be present in an emergency department environment, and familiarization and training is essential for all staff who will be the frontline providers of thrombolytic treatment—most often nurses, doctors in training, or non-specialist medical staff. Pharmacists are also closely involved in training, putting in place clear instructions for correct dosing and drug preparation, and in some regions also in drug preparation. Regions that have introduced tenecteplase as standard stroke thrombolysis have done so after education to minimize the potential for dose errors arising from the selection of the incorrect tenecteplase pack.^25^ In many regions, the inclusion in tenecteplase packs for acute MI treatment of a weight-graduated syringe and diluent serves as a possible warning of incorrect drug pack selection. Telethrombolysis support that includes video may further mitigate risks by allowing experienced stroke physicians to visually check that the correct tenecteplase pack has been selected prior to reconstitution and administration. Volumes of tenecteplase for stroke reconstituted per license guidelines should be between 3 and 5 mL.

## Complications of thrombolysis

Some physicians express anxieties around potential bleeding complications that can no longer be managed by stopping an infusion. Intracranial hemorrhage, however, seldom manifests clinically during alteplase infusion: in a large real-world series from the United States, only 1 of 511 patients treated with alteplase experienced deterioration due to intracerebral hemorrhage within 1 h of the bolus being administered.^26^ Since alteplase is associated with prolonged derangement of hemostasis (reduced fibrinogen and plasminogen being seen 24 h after administration^27^), stopping an alteplase infusion is unlikely to significantly modify either systemic or intracranial bleeding complications. Tenecteplase was not associated with significant hemostasis derangements in a small sub-study of stroke patients,^27^ although notably this has not translated into reduced incidence of intracerebral or systemic bleeding complications reported in RCTs compared to alteplase for acute ischemic stroke.

## Indications for thrombolysis outside its current license

The recent findings of TEMPO-2 showed no benefit from tenecteplase 0.25 mg/kg in patients with non-disabling stroke even in the presence of intracranial vessel occlusion.^28^ Randomized controlled trial of TNK-tPA versus standard of care for minor ischemic stroke with proven occlusion (TEMPO-2) findings for tenecteplase are consistent with non-inferiority of dual antiplatelet therapy compared to alteplase in a similar population in the Chinese Antiplatelet vs R-tPA for Acute Mild Ischemic Stroke (ARAMIS) trial,^29^ and also with lack of benefit of alteplase compared to control in the Potential of rtPA for Ischemic Strokes With Mild Symptoms (PRISMS) trial, albeit interpretation of PRISMS is severely limited by its early termination and much smaller sample size than planned.^30^

Is there a case for retention of alteplase for guideline-accepted acute stroke indications that lie outside the recent head-to-head trials? The two potential indications are late time window intervention 4.5–9 h from onset based on brain perfusion as defined in the EXTEND trial,^31^ and unknown onset or wake-up stroke based on either the WAKE-UP diffusion-FLAIR mismatch profile^32^ or the EXTEND perfusion-based profile. In reality, there is a substantial overlap between these groups, and eligibility for treatment depends on brain imaging signatures of tissue viability rather than relying on medical history of timing. Neither indication is covered by regulatory approvals and therefore represents off-label use, albeit supported by high-quality RCTs, individual patient data meta-analyses (IPDMAs), and international guidelines.^33^ Ideally RCT data that compare tenecteplase against alteplase would ultimately support a recommendation in favor of change to tenecteplase in these patient groups; however, there are no relevant published trials currently, and an adequately powered non-inferiority trial would be a significant undertaking given the much lower frequency of these clinical presentations.

Is it justified to retain alteplase pending head-to-head RCTs in these specific clinical situations? In the absence of direct comparative data, trials of tenecteplase against control offer relevant information on safety and efficacy.

**Figure 2.**
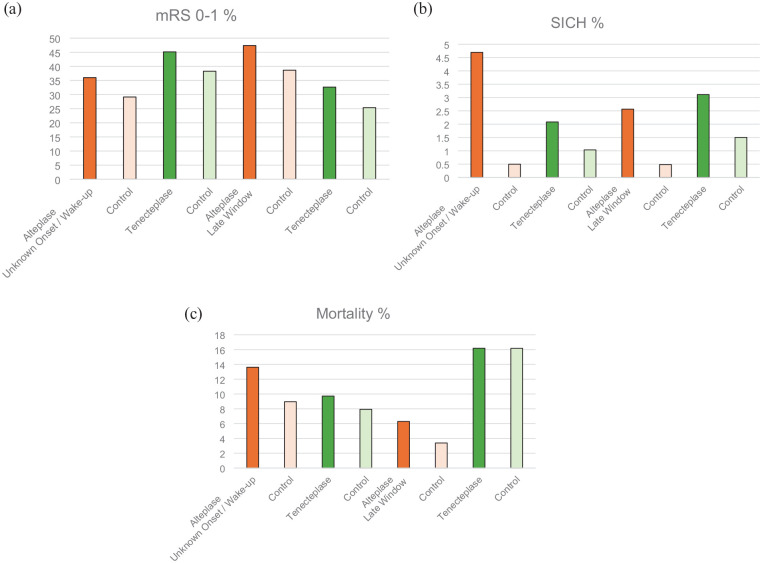
Percentage of outcomes for A mRS 0–1 B SICH and C mortality for trials comparing alteplase against control (orange; data taken from individual patient data meta-analyses) and tenecteplase against control (green; unknown onset / wake-up data from TWIST, late window data combined figures from TIMELESS and TRACE-3).

The TWIST trial undertaken in patients with wake-up stroke based on the absence of extensive hypoattenuation on non-contrast CT (NCCT) reported a non-significant 7% increase in mRS 0–1 outcomes favoring tenecteplase 0.25 mg/kg and odds ratios favoring tenecteplase on all functional outcomes.^34^ Lack of statistical significance may be attributable to smaller than planned sample size due to early trial termination.^34^ The magnitude of the effect size and safety profile is similar to that observed in WAKE-UP, EXTEND, and the individual patient data meta-analysis of thrombolysis for stroke of unknown onset time (Table 2 and Figure 2).

**Table 2.** Late window and wake-up thrombolytic trials in stroke, showing effect sizes for excellent clinical outcome (mRS of 0 or 1 at Day 90), SICH rates, and mortality.

Agent	Trial	Imaging selection	mRS 0–1	SICH	Mortality
Alteplase	IPDMA (EXTEND, ECASS-4, EPITHET)^35^	CTP (EXTEND), MRI diffusion—perfusion mismatch (ECASS-4, EPITHET)	76/211 (36%) alteplase versus 58/199 (29%) placebo	10//213 (5%) alteplase versus 1/201 [< 1%] placebo	29/213 (14%) alteplase versus 18 /201 (9%) placebo
	IPDMA—EOS (WAKE-UP, EXTEND, THAWS, ECASS-4)^36^	Diffusion-FLAIR mismatch (WAKE-UP), MRI diffusion-perfusion mismatch (ECASS-4), CTP mismatch (EXTEND)	199/420 (47%) alteplase versus 160/414 (39%) control	11/429 [3%] alteplase versus 2/414 (< 1%) control	27 /429 (6%) alteplase versus 14 /414 (3%) control
Tenecteplase	TWIST^34^	NCCT	130/288 (45%) tenecteplase versus 111/290 (38%) control	6/288 (2%) tenecteplase versus 3/290 (1%) control	28 / 288 (10%) tenecteplase versus 23/290 (8%) control
	TIMELESS^37^	CTP	73/226 (32%) tenecteplase versus 61/229 (27%) control	7/218 (3.2%) tenecteplase versus 5/214 (2.3%) control	43/218 (19.7%) tenecteplase versus 43/218 (19.7%) control
	TRACE-3^38^	CTP	87/264 (33%) tenecteplase versus 61/252 (24%) control	8/264 (3%) tenecteplase versus 2/252 (0.8%) control	35/264 (13)% tenecteplase versus 33/252 (13%) control

In the late time window for stroke of known onset time, the TIMELESS trial used CT Perfusion (CTP) selection slightly more restrictive than that of EXTEND (requiring a penumbra:core ratio > 1.8 rather than the > 1.2 required in EXTEND) and an even longer time window of between 4.5 and 24 h from onset.^37^ TIMELESS did not show significant benefit for its primary outcome of median mRS at Day 90, but had very high rates of endovascular thrombectomy (77% of all enrolled patients) and very short thrombolysis bolus-to arterial puncture times in both trial arms (medians of 15 and 17 min) that would be expected to minimize any difference between treatment arms. Despite this, TIMELESS reported numerical benefit in favor of independent recovery with tenecteplase, and safety data very similar to that observed for alteplase in late time windows.^37^

Finally, TRACE-3 investigated the tenecteplase biocopy molecule available in China in patients with a CTP profile of viable tissue using the DEFUSE-3 definition also employed in TIMELESS (minimum Tmax > 6 s volume 15 mL, core volume defined by rCBF < 30% < 70 mL, ratio of penumbra to core volume > 1.8)^39^ treated between 4.5 and 12 h from onset who did not proceed to thrombectomy. The trial showed superiority of tenecteplase 0.25 mg/kg over control, with a magnitude of effect very similar to that observed with alteplase in EXTEND.^38^ Since data from TRACE-2,^3^ the head-to-head comparison of the tenecteplase biocopy with alteplase in the 4.5 h window, closely mirror the non-inferiority findings reported from trials that employed the globally available European-manufactured tenecteplase, it is unlikely that the biocopy tenecteplase molecule differs in a clinically meaningful way.

Formal individual patient data meta-analysis of late window or unknown onset stroke tenecteplase trials may in due course provide more robust estimates of effect size and outcome, but the key efficacy and safety outcomes closely match those seen with alteplase in prior trials (Table 2 and Figure 2). In addition, given the robust evidence of non-inferior efficacy and similar safety profile for tenecteplase 0.25 mg/kg compared to alteplase in the 4.5 h time window, there is no compelling biological basis to expect a different profile in these other patient groups. While the European Stroke Organization guideline group considered the evidence base prior to recent trials to be too weak to support the use of tenecteplase over alteplase in stroke of unknown onset time, consensus expert opinion even at that time unanimously favored tenecteplase as a reasonable alternative among patients meeting imaging criteria (MRI or CTP-based).^17^

## Conclusion

There are clear potential risks of dose and administration errors as a consequence of retaining alteplase in emergency care environments that will probably also have two distinctly packaged tenecteplase preparations for different indications with different doses (stroke and MI): instances have been reported of confusion arising between alteplase and tenecteplase dose schedules (e.g. https://www.hdc.org.nz/decisions/search-decisions/2016/13hdc01676/). Communicating nuances of patient selection from stroke experts to non-expert medical or nursing staff may be challenging, especially in telethrombolysis settings. The same staff are likely to use thrombolytic drugs for stroke only infrequently, and are likely to have some exposure to uses for other indications. While a further direct comparison of alteplase with tenecteplase in specific off-label uses would be scientifically rigorous, in the absence of such a trial, there is a strong safety-based rationale for a single universal switch to tenecteplase 0.25 mg/kg for all acute ischemic stroke thrombolysis including off-label use in wake-up / unknown onset and late time window cases who fulfill the MRI or perfusion-based imaging criteria employed in relevant clinical trials. This ensures a clear and unequivocal training message that can be delivered to all health care staff involved in acute stroke treatment.
